# The effect of resource loss on depression and peritraumatic distress during the early period of the COVID-19: considering the pandemic-situational and social context

**DOI:** 10.1186/s12889-023-15628-5

**Published:** 2023-04-25

**Authors:** Yubin Lee, Yoorim Hyun, Myoungsoon You, Heeyoung Lee, Jin-Ok Han, Suin Seo

**Affiliations:** 1 Department of Public Health, Graduate School of Public Health, Seoul National University, Seoul, 08826 Korea; 2 Gyeonggi Public Health Policy Institute, Seongnam, 13605 Korea; 3 Public Health Care Headquarters, Bundang Seoul National University Hospital, Seongnam, 13620 Korea

**Keywords:** COVID-19, Conservation of resources theory, Mental health, Depression, Peritraumatic distress

## Abstract

**Background:**

The public experienced loss of resources, including their health and property during the COVID-19 pandemic. The Conservation of Resources (COR) theory is a useful tool to explain the effect of resource loss on mental health. This paper examines the effect of resource loss on depression and peritraumatic distress considering the situational and social context of the COVID-19 pandemic applying COR theory.

**Methods:**

An online survey was conducted for Gyeonggi residents when the second wave of COVID-19 in South Korea declined (5 October to 13 October 2020); 2,548 subjects were included in the hierarchical linear regression analysis.

**Results:**

COVID-19 infection-related experiences, resource losses (e.g., financial burden, deterioration of health, and decline of self-esteem), and fear of stigma were related to elevated levels of peritraumatic distress and depression. Risk perception was associated with peritraumatic distress. Reduced income or job loss were related to depression. Social support was a protective factor for mental health.

**Conclusions:**

This study suggests that we need to focus on COVID-19 infection-related experiences and loss of daily resources in order to understand mental health deterioration during the COVID-19 pandemic. Moreover, it is important to monitor the mental health of medically and socially vulnerable groups and those who have lost resources due to the pandemic and to provide them with social support services.

## Background

During the pandemic, many individuals underwent major changes in their daily life. For example, as a result of the closure of public facilities, such as schools and daycare centers, daily routines were disrupted and educational opportunities and professional experiences diminished [1]. Some lost their jobs and social networks due to physical distancing and lockdowns [2]. These changes affected people’s mental health. In many countries, including Sweden, the United States, and the United Kingdom, the prevalence of depression has increased significantly compared to pre-pandemic levels [3]. In meta-analyses of the general population, the pooled prevalence of posttraumatic symptoms and psychological stress early in the COVID-19 pandemic was 23.9% and 24.8%, respectively [4].

The Conservation of Resources Theory (COR theory) has been a useful framework to understand mental health deterioration in times of crisis [5, 6]. COR theory focuses on resources to understand psychological stress. According to the theory, people endeavor to obtain, maintain, or protect their resources and they experience psychological distress when such resources are threatened or lost [5]. The resources include object resources (e.g., food and home), conditions (e.g., marital status and employment), personal characteristics (e.g., self-esteem and optimism), energies (e.g., time and money), and such resources aggregate in ‘resource caravans’ [5]. Resource loss is disproportionately more salient than resource gain, but resource gain becomes more important in the context of resource loss because it buffers the aversive effect of resource loss [7].

Traumatic events like disasters cause a rapid loss of resources and high levels of stress because they often occur unexpectedly, affect the most valuable resources, and make it hard for people to cope with the existing resources [6, 8]. Resource loss during the COVID-19 pandemic was associated with negative psychological outcomes, such as COVID-19-related post-traumatic symptoms, stress, loneliness, and emotional distress [9–11]. To be specific, psychological distress due to COVID-19 was positively related to the loss of financial resources, family resources, control over the future, fun, and social resource [10]. Personal resilience and social support were protective factors for mental health problems [9, 12]. There were also differences in the level of psychological distress based on socioeconomic status, including gender, race, and ethnicity [9, 11].

In the context of an epidemic outbreak, risk perception and infection-related experience can be indicators of threat or loss of health. There are a few studies that denoted the risk perception (or perceived risk) of COVID-19 as a threat to resources, but the studies focused mostly on workers [13, 14]. Additionally, few studies applied COR theory to address infection-related experience as a risk factor for mental deterioration [15]. Therefore, it is necessary to consider variables that reflect the context of the COVID-19 outbreak, such as risk perception and infection experience to assess the impact of resource loss on mental health. Moreover, mental health is affected by social factors as well as personal factors [16]. To be specific, social support is one of the most important social resources in COR theory, and it serves as a major reservoir for resources other than personal resources [17]. Several studies revealed that social support buffered the adverse effect of resource loss during the pandemic [12, 18]. In addition, other factors such as neighborhood social relations and stigma related to infection were associated with mental health, but these factors were rarely reflected in the previous studies applying the COR theory during the pandemic [16, 19, 20].

In the present study, we investigated the impact of resource losses during the COVID-19 pandemic on mental health, through the lens of COR theory. Furthermore, we took situational and social factors into account. It was hypothesized that (1) sociodemographic factors and health status (2) risk perception and infection-related factors (i.e., situational factors) (3) resource loss and (4) social factors (e.g., social support and stigma) will be associated with a negative mental health outcome.

## Methods

### Participants and procedures

In this study, we adopted a cross-sectional survey design to assess Gyeonggi residents' mental health and related resources during the COVID-19 pandemic. Gyeonggi has the largest population among the 17 administrative districts and the largest number of new and cumulative confirmed cases in South Korea (as of April 2022) [21, 22]. At the time of the survey, approximately 18.9% of the cumulative confirmed cases in Korea were reported from Gyeonggi [23]. The survey was conducted online over eight days (5 October to 13 October 2020), when the second wave of the COVID-19 pandemic in South Korea declined. Health authorities announced a two-week “special prevention period” (28 September to 11 October 2020) to strengthen prevention measures due to the Chuseok holiday [24].

Respondents were recruited from survey panel members of Hankook Research, one of the largest research companies in South Korea with a panel of 1.69 million. Adults (18 years and over) in Gyeonggi were recruited using proportional and quota sampling methods based on age and sex. The survey Uniform Resource Locator (URL) containing the study purpose and consent statement was sent via text message and e-mail. A total of 2,548 people participated in the survey.

### Measures

#### Dependent variables: peritraumatic distress and depression

The public’s mental health was assessed using two instruments. The 13-item peritraumatic distress inventory (PDI) was used to measure the level of peritraumatic distress, or the physiological, emotional, and cognitive responses experienced during and shortly after a traumatic event [25, 26]. The PDI is a self-report questionnaire consisting of two factors: negative emotions (e.g., “I felt helpless to do more”) and perceived life threat and bodily arousal (e.g., “I had physical reactions like sweating, shaking, and my heart pounding”). The PDI was demonstrated to be internally consistent and have good test-retest reliability as well as good convergent and divergent validity [25]. We used the Korean version of the PDI, which was translated into Korean by public health professionals who are fluent in both English and Korean, using the back-translation process [27]. Participants rated the items using a 5-point Likert-type scale (0 = “not at all true” to 4 = “extremely true”). The evaluations were summed to calculate the total score, with a higher total score indicating a highly distressed status. The PDI had excellent internal consistency in this study (Cronbach’s α = 0.92).

The Patient Health Questionnaire-9 (PHQ-9) is a reliable and valid tool for measuring the severity of depression and is widely used in primary care settings [28, 29]. We used the Korean version of the PHQ-9, which was translated by psychiatric professionals and demonstrated to be reliable and valid [30]. Participants evaluated how often they had been bothered by the presented problems (9 items) over the past two weeks, using a 4-point Likert-type scale (0 = “not at all,” 1 = “several days,” 2 = “more than half the days,” 3 = “nearly every day”). The ratings were added together, and a higher total score indicates more severe depression. The PHQ-9 showed excellent internal consistency in this study (Cronbach’s α = 0.91).

#### Independent variables

##### Sociodemographic factors and health status

Sociodemographic factors included gender, age, education level, marital status, and monthly household income (KRW). Participants were also asked to rate their health status (1: Very bad – 5: Very bad).

##### Risk perception and infection-related factors

Risk perception of COVID-19 infection was measured with two items following the previous research [31, 32]. Respondents rated the perceived susceptibility and the perceived severity of COVID-19 infection using a 5-point Likert-type scale (1 = “not at all” to 5 = “very much”), and the average of the evaluations for the two items was calculated. The greater the score, the higher the risk perception.

Additionally, we asked participants if they or someone close to them had been infected or had been isolated or quarantined due to COVID-19 (0 = “not experienced” and 1 = “experienced.”)

##### Resources Loss and change in employment status

The item scale was constructed to assess the extent of resource losses due to the COVID-19 pandemic. The Conservation of Resource-Evaluation(COR-E) questionnaire, a tool to measure resource loss developed by Hobfoll, has four constructs: material, personal, energy, and interpersonal losses [33]. Such constructs were used to guide item generation and 12 items were selected for each of the four domains of the COR theory. Examples include “It was hard for me to buy necessities,” “It negatively affected my self-esteem,” and “It had a negative influence on my physical health.” The items were translated into Korean by the public health professionals of the research team and reviewed by another public health expert, all of whom were fluent in both Korean and English. The participants responded to the items regarding how much COVID-19 has affected their resources using a 5-point Likert-type scale (0 = “not at all” to 4 = “very much”). The higher the score, the greater the degree of resource loss caused by the COVID-19 pandemic.

Furthermore, respondents answered the question “Have you had any changes in employment status and wages due to COVID-19?” (1 = “I got the same wages as before COVID-19,” 2 = “I did not lose my job, but my wages declined,” 3 = “I did not lose my job, but had unpaid leave,” 4 = “I lost my job,” 5 = “economically inactive population”). We classified the responses into four groups: ‘economically inactive,’ ‘maintained employment status and income,’ ‘maintained employment status but reduced income,’ and ‘lost job.’

##### Social variables

Social support was evaluated by the number of people, other than family, to whom participants could turn for urgent help if they had to be isolated (or quarantined) due to COVID-19 infection or close contact with a confirmed case ( 0 = “no one,” 1 = “1-2 people,” 2 = “3-4 people,” 4 = “more than 5 people”) [32]. The more persons they had access to for assistance, the higher the level of social support.

Fear of social stigma was evaluated with three items, following Yoon, You, and Shon [27]: [I am afraid that…] “if I become a confirmed COVID-19 patient, I will be criticized or disadvantaged based on this fact”, (2) “if there are confirmed patients in my neighborhood, my neighborhood will be criticized or disadvantaged based on this fact”, or (3) “if there are confirmed patients in my group (e.g., workplace and religion), my group will be criticized or disadvantaged based on this fact.” Participants indicated their level of agreement with the statements using a 5-point Likert-type scale (1 = “not at all” to 5= “very much”), and the average ratings for three items were calculated. The inter-item reliability was acceptable (Cronbach’s α = 0.79).

Trust in the neighborhood is the belief that members of the neighborhood are willing to comply with COVID-19 prevention guidelines, have sufficient capacity to deal with this public health emergency, and have the resilience to recover. It was assessed by asking respondents how much they agree with six statements such as “People in my neighborhood follow personal preventive measures well,” “My neighborhood will overcome the crisis and recover effectively even if suffering from infectious diseases.” Participants responded using a 5-point Likert-type scale (1 = “strongly disagree” to 5 = “strongly agree”). The average ratings for six items were then calculated. The inter-item reliability was good (Cronbach’s α = 0.89).

### Data analysis

First, descriptive analysis was performed to present the sociodemographic characteristics, COVID-19-related resources, and mental health status of the participants. The results were reported either as frequency (percentage, %) or mean (M) and standard deviation (SD).

Second, a four-step hierarchical linear regression was conducted to estimate the associations between resources and mental health during COVID-19. In the first step, we included sociodemographic factors and health status as potential confounding factors affecting mental health (Model 1). In the second step, we added risk perception and COVID-19 infection-related experiences to examine the effects of life threats during the pandemic on mental health (Model 2). In the third step, we added resource loss and change in employment status to examine the impact of resource loss and threats to livelihood during the COVID-19 pandemic on mental health (Model 3). In the final step, we added social variables (i.e., social support, fear of social stigma, and trust in the neighborhood) (Model 4). The variance inflation factor (VIF) was applied to detect multicollinearity in each hierarchical model. As there was no VIF value exceeding 10, we determined that there was no multicollinearity. Statistical analyses were conducted using the R version 4.1.1 software (R Foundation for Statistical Computing, Vienna, Austria).

### Ethical considerations

The study was approved by the IRB of Seoul National University of Bundang Hospital (B-2005-615-303).

## Results

### Sociodemographic characteristics

Sociodemographic characteristics are presented in Table 1. Among the 2,548 participants, there were 1,273 men (50.0%) and 1,275 women (50.0%). The average age of the participants was 44.8 years old. The majority of the participants received at least some college education (78.6%), and some had less than a high school education (21.4%). More participants had a spouse (64.4%) than were unmarried, divorced, or bereaved (35.6%). The most common monthly household income was over KRW 6 million (36.3%), followed by KRW 2-3.99 million (28.3%), and KRW 4-5.99 million (27.4%). Most participants reported their subjective health status as moderate (48.7%) or good (42.2%).

**Table 1:** Sociodemographic characteristics of participants

	**N (2,548)**	**% (100)**
**Gender**		
Men	1273	50.0
Women	1275	50.0
**Age**	*M* = 44.8 (*SD* = 13.58)	
18-29	447	17.5
30-39	479	18.8
40-49	584	22.9
50-59	555	21.8
≥ 60	483	19.0
**Education level**		
Less than high school	545	21.4
College and above	2003	78.6
**Marital status**		
Unmarried/Divorced/Bereaved	908	35.6
Married	1640	64.4
**Monthly household income **^**a**^	*M* = 5.64 (*SD* = 2.38)	
< 2.00	204	8.0
2.00-3.99	721	28.3
4.00-5.99	698	27.4
≥ 6.00	925	36.3
**Subjective health status**	*M* = 3.39 (*SD* = 0.75)	
Bad	232	9.1
Moderate	1241	48.7
Good	1075	42.2

^a^million KRW (KRW 1 = USD 0.0086)

### COVID-19-related variables and mental health status

The results of descriptive analysis on COVID-19-related variables and mental health status are presented in Table 2. The average PDI score was 22.63 (SD = 9.87). Most of the participants (66.2%) required a checkup after several weeks, and 27.0% required immediate care and follow-up. The average PHQ-9 score was 6.97 (*SD* = 6.04), and 28.2% of the participants were moderately to severely depressed.

**Table 2:** COVID-19-related variables and mental health

	***N* (2,548)**	**% (100)**	**Range**	***M* (*SD*)**
Peritraumatic distress (PDI)			0-52	22.63 (9.87)
Normal (PDI < 7)	172	6.8		
Requires a checkup in time (PDI: 7–28)	1687	66.2		
Requires immediate care and follow-up (PDI > 28)	689	27.0		
Depression (PHQ-9)			0-27	6.97 (6.04)
Normal to mild depression (PHQ-9 < 10)	1829	71.8		
Moderate–severe depression (PHQ-9 ≥ 10)	719	28.2		
Risk perception			1-5	3.43 (0.57)
COVID-19 infection-related experiences				
Yes	374	14.7		
No	2174	85.3		
Resources loss			0-4	1.97 (0.68)
Change in employment status				
Economically inactive	768	30.1		
Maintained employment status and income	932	36.6		
Maintained employment status but reduced income	630	24.7		
Lost job	218	8.6		
Social support			1-4	2.29 (0.81)
No one	335	13.2		
1-2 people	1361	53.4		
3-4 people	619	24.3		
More than 5 people	233	9.1		
Fear of stigma			1-5	3.38 (0.85)
Trust in the neighborhood			1-5	3.65 (0.61)

The average risk perception score was 3.43, which was higher than moderate (score = 3). Approximately 14.7% of participants or their close acquaintances were infected with COVID-19 or had to be isolated or quarantined. Regarding employment status, 36.6% of the participants maintained their jobs and income, 24.7% kept their jobs but their incomes declined, and 8.6% lost their jobs. The majority of participants had at least one person they could ask for help with during self-isolation or self-quarantine, but 13.2% of participants had no one to ask for help. The average score of fear of social stigma was 3.38 (SD = 0.85), which indicates that participants felt stigma-related fear that was higher than moderate level (score = 3). The average trust in the neighborhood score was 3.65 (SD = 0.61), which was higher than moderate (score = 3).

### Hierarchical linear regression

#### The influence of COVID-19-related variables on peritraumatic distress

Table 3 shows the results of a four-step hierarchical linear regression analysis for peritraumatic distress. In model 1, sex, age, marital status, and subjective health status were significant predictors of peritraumatic distress (*R*^*2*^ = .055, *F* (5,2542) = 29.71, *p* < .001). Peritraumatic distress was higher among females and those that were younger, married, and less healthy. Model 2 shows that risk perception and COVID-19 infection-related experiences were positively related to peritraumatic distress (*R*^*2*^ = .140, *F* (7,2540) = 58.91, *p* < .001) and explained 8.5% of the variance in this variable. Model 3 indicates that resource loss during COVID-19 predicted peritraumatic distress, whereas a change in employment status did not (*R*^*2*^ = .47, *F* (11, 2536) = 204.5, *p* < .001). The variables accounted for 33.0% of the variance in peritraumatic distress. Marital status and age were no longer predictors of peritraumatic stress in this model. In Model 4, we added variables related to social resources, which explained 2.8% of the total explained variance. Social support and fear of social stigma were predictors of peritraumatic distress after controlling for other variables (*R*^*2*^ = .498, *F* (14, 2533) = 179.6, *p* < .001). Social support was negatively associated with peritraumatic distress and social stigma was positively associated with peritraumatic distress. In the final model (Model 4), high-risk perception, COVID-19 isolation/quarantine experiences, loss of resources, and fear of social stigma were risk factors, and health and social support were protective factors of peritraumatic stress during the COVID-19 pandemic.

**Table 3:** Hierarchical linear regression for predicting peritraumatic distress

**Variable (PDI)**	**Model 1**		**Model 2**		**Model 3**		**Model 4**	
	***B* (*SE*)**	**β**	***B* (*SE*)**	**β**	***B* (*SE*)**	**β**	***B* (*SE*)**	**β**
Sex (ref=men)	1.299 (0.382)***	.066	1.182 (0.366)**	.060	0.656 (0.296)*	.033	0.308 (0.293)	.016
Age	-0.063 (0.017)***	-.087	-0.041 (0.016)*	-.057	-0.007 (0.013)	-.010	-0.017 (0.012)	-.024
Marital status (ref=Unmarried/Divorced/Bereaved)								
Married	1.691 (0.482)***	.082	1.583 (0.461)***	.077	0.650 (0.363)	.032	0.450 (0.354)	.022
Monthly household income	-0.111 (0.083)	-.027	-0.094 (0.079)	-.023	0.018 (0.063)	.004	0.046 (0.062)	.011
Subjective health status	-2.707 (0.257)***	-.206	-1.848 (0.252)***	-.141	-0.871 (0.200)***	-.066	-0.936 (0.197)***	-.071
Risk perception			4.633 (0.332)***	.265	2.326 (0.268)***	.133	1.654 (0.267)***	.095
COVID-19 infection-related experiences			3.647 (0.521)***	.131	2.135 (0.412)***	.077	2.091 (0.401)***	.075
Resources loss					8.778 (0.230)***	.600	7.971 (0.236)***	.545
Change in employment status (ref=economically inactive population)								
Maintained employment status and income					-0.283 (0.368)	-.014	-0.529 (0.359)	-.026
Maintained employment status but reduced income					0.319 (0.400)	.014	0.187 (0.389)	.008
Lost job					-0.117 (0.562)	-.003	0.123 (0.548)	.003
Social support							-0.559 (0.179)**	-.046
Fear of social stigma							2.036 (0.180)***	.175
Trust in neighborhood							0.369 (0.234)	.023
*F (p-value)*	29.71 (<.001)		58.91 (<.001)		204.5 (<.001)		179.6 (<.001)	
*R*^*2*^*(adj.R*^*2*^	.055 (.053)		.140 (.137)		0.470 (.468)		0.498 (.495)	
*ΔR*^*2*^			.085***		.330***		.028***	

^*^ p < .05, ** p < .01, *** p < .001

### The influence of COVID-19-related variables on depression

Hierarchical linear regression analysis for depression is presented in Table 4. The results of Model 1 show that men and those of a younger age, with a low monthly household income, and in poor health were more likely to have depression (*R*^*2*^ = .096, *F* (5, 2542) = 53.86, *p* < .001). In Model 2, adding risk perception and COVID-19 infection-related experiences accounted for 3.9% of the change. Individuals who had higher levels of risk perception and COVID-19 infection-related experiences (themselves or close acquaintances) showed higher levels of depression (*R*^*2*^ = .135, *F* (7, 2540) = 56.59, *p* < .001). Model 3 indicates that resource loss and change in employment status were predictors of and accounted for 21.3% of the variance in depression (*R*^*2*^ = .348, *F* (11, 2536) = 122.9, *p* < .001). Depression was higher as more resources were lost during COVID-19. Individuals with reduced income or who were unemployed had higher levels of depression than the economically inactive group. Model 4 model shows that social support was negatively associated with depression and fear of stigma was positively associated with depression (*R*^*2*^ = .361, *F* (14, 2533) = 102.3, *p* < .001). Social resource variables explained 1.3% of the total explained variance. The final model indicates that sex, age, marital status, health status, COVID-19 infection-related experiences, resource loss, employment status change, social support, and fear of social stigma were significant predictors of depression during the COVID-19 pandemic.

**Table 4:** Hierarchical linear regression for predicting depression

**Variable (PHQ-9)**	**Model 1**		**Model 2**		**Model 3**		**Model 4**	
	***B* (*SE*)**	**β**	***B* (*SE*)**	**β**	***B* (*SE*)**	**β**	***B* (*SE*)**	**β**
Sex (ref=Men)	0.820 (0.229)***	.068	0.806 (0.224)***	.067	0.661 (0.201)**	.055	0.429 (0.203)*	.035
Age	-0.083 (0.010)***	-.187	-0.072 (0.010)***	-.162	-0.057 (0.009)***	-.128	-0.058 (0.009)***	-.130
Marital status (ref=Unmarried/Divorced/Bereaved)								
Married	-0.125 (0.288)	-.010	-0.132 (0.283)	-.010	-0.535 (0.247)*	-.042	-0.577 (0.245)*	-.046
Monthly household income	-0.155 (0.050)**	-.061	-0.153 (0.049)**	-.060	-0.100 (0.043)*	-.039	-0.067 (0.043)	-.026
Subjective health status	-1.749 (0.154)***	-.217	-1.445 (0.155)***	-.180	-0.998 (0.136)***	-.124	-0.941 (0.136)***	-.117
Risk Perception			1.588 (0.204)***	.149	0.459 (0.182)*	.043	0.266 (0.184)	.025
COVID-19 infection-related experiences			2.282 (0.320)***	.134	1.492 (0.280)***	.087	1.517 (0.277)***	.089
Resources loss					4.063 (0.156)***	.454	3.763 (0.163)***	.420
Change in employment status (ref=economically inactive population)								
Maintained employment status and income					0.192 (0.249)	.015	0.135 (0.248)	.011
Maintained employment status but reduced income					1.226 (0.271)***	.088	1.194 (0.269)***	.085
Lost job					1.443 (0.381)***	.067	1.532 (0.378)***	.071
Social support							-0.693 (0.124)***	-.093
Fear of social stigma							0.564 (0.125)***	.079
Trust in neighborhood							-0.175 (0.162)	-.018
*F (p-value)*	53.86 (<.001)		56.59 (<.001)		122.9 (<.001)		102.3 (<.001)	
*R*^*2*^*(adj.R*^*2*^	.096 (.094)		.135 (.133)		.348 (0.345)		.361 (0.358)	
*ΔR*^*2*^			.039***		.213***		.013	

^*^ p < .05, ** p < .01, *** p < .001

## Discussion

This study examined the effect of resource loss on mental health during the COVID-19 pandemic by applying the COR theory and considering the situational (risk perception and COVID-19 infection-related experience) and social (social support, fear of social stigma, trust in neighborhood) context. There are several notable findings in this study.

First, COVID-19 infection-related experiences were associated with peritraumatic distress and depression, which is consistent with the results of previous studies [27, 34]. COVID-19 threatens health, daily functioning, and safety which are related to basic needs for humans [35, 36]. In addition to fear of death and the physical discomfort caused by disease, COVID-19 patients are exposed to stressors such as guilt, concerns about the health of loved ones, privacy violations, and social stigma [37, 38]. Likewise, a quarantine experience during the COVID-19 outbreak is related to fear of infection, frustration and boredom, social rejection, and financial problems [39]. In order to mitigate the consequences of COVID-19 quarantine and isolation, officials should restrict the length of isolation or quarantine as short a time as possible based on scientific evidence, and provide adequate resources such as reliable information and basic supplies [39]. In addition, after controlling for other variables, the effect of risk perception on distress persisted in this study. Even if risk perception decreased with new variants and the increased availability of vaccines, perceived risk can still be high for people who are more likely to be exposed to the virus and have more severe effects (e.g., patients with high-risk diseases or healthcare workers) [40, 41]. Trust in government and medical professionals is related to risk perception, so governments should maintain their accountability by improving preparedness for and responses to infectious diseases, which includes ensuring sufficient healthcare resources and funding [42, 43]. The leaders should communicate empathically addressing the feelings and hardships of the public to build trust and rapport [44].

Second, resource loss during the COVID-19 pandemic had a significant effect on mental health. Greater levels of peritraumatic distress and depression were associated with greater losses of resources. As people already lacking in resources are more likely to experience loss spirals, socially vulnerable groups like low-income and ethnic minorities are more likely to have suffered severe psychological damage during the pandemic than those with abundant resources [5, 45, 46]. Moreover, those who lost resources and opportunities due to COVID-19 but are not socially disadvantaged, such as young job seekers or family caregivers, are more likely to experience mental distress [47, 48]. Especially, job loss and financial hardships that ensue as a result, have been one of the biggest issues during the COVID-19 pandemic. In a 2022 Pew Research Center survey, 71% of Americans considered strengthening the economy a major policy priority, while 60% prioritized managing the COVID-19 outbreak [49]. In this study, change in employment status during the pandemic had a significant impact on depression levels. Unemployment is associated with the deterioration of positive personal resources, such as resilience, optimism, and self-efficacy, and impacts other conditions (e.g., seniority and tenure at work, energy resources) [50, 51]. Delivering integrated mental health and employment services through integration in organizations and coordination is necessary to support mental health [3, 52]. This includes providing jobseekers with job-search assistance, counseling, and training opportunities to facilitate their return to the workforce [3].

Third, social support plays an important role in coping with stressful situations, as noted in previous studies on stress [53]. Social connectedness and social support during or after a traumatic event are associated with lower levels of mental health problems [54, 55]. Generally, individuals feel comfortable seeking help from family and friends, but social interactions with these people may have been reduced during the COVID-19 pandemic [56, 57]. Since it is difficult to maintain face-to-face social relationships during infectious disease outbreaks, social support systems, such as remote consultations or social prescribing services, should be utilized [58].

Furthermore, fear of social stigma was a risk factor for peritraumatic distress and depression. When responding to a highly uncertain and previously unknown risk such as COVID-19, people may fear the unfamiliar peril and stereotype or discriminate against those deemed to be related to the risks [59]. During the COVID-19 pandemic, stigma was directed against suspected and confirmed patients, dead people and their families, healthcare workers, and out-group members (e.g., migrants, and people of different races or religions) [20]. Even though the fear of the infection and the stigma against COVID-19 patients may decrease due to the increased knowledge about the COVID-19 and acquisition of immunity from vaccines, the stigma would not be eradicated. In previous research, the SARS-associated stigma of SARS victims was maintained and reconstructed even after the epidemic had been over, leaving SARS victims psychologically distressed [60]. Furthermore, when the stigma of infection intersects with existing prejudices in society (such as racism), they create a social stigma that can endure long after an outbreak of an epidemic [61]. Therefore, the government should follow up mental health of stigmatized people during the pandemic and provide them with social support services [60]. Moreover, it is necessary to address broader social stigmas in order to change discriminatory social norms (e.g., building safe and inclusive environments by laws and policies) [61, 62].

This study has the following limitations. First, since it was conducted as a cross-sectional study, it is difficult to infer the causal relationship between variables. Second, the survey was conducted in the early period of the COVID-19 pandemic, before the emergence of diverse SARS-CoV-2 variants [63]. A follow-up study may be needed to capture the effects of these virus variants on mental health outcomes. Finally, we used the average score of resource losses in the analysis, so it is difficult to know what kind of resource losses (e.g., physical health, money, or self-esteem) had the most severe impact on mental health deterioration.

## Conclusions

This study investigated the effect of the loss of resources during COVID-19 on mental health, using the lens of COR theory. We additionally explored the impact of COVID-19-related situational and social factors. The results indicate that COVID-19 itself threatened the physical and mental health of the public. Improving preparedness and responses to other new infectious diseases remains important. Since the resource loss during COVID-19 resulted in negative mental health outcomes, it is important to investigate what kinds of resources need to be gained to mitigate this effect. It is necessary to monitor the mental health status of people who have previously lacked resources (e.g., medically or socially vulnerable populations) or who lost enormous resources due to the pandemic and provide them with social support programs, including economic support and community referral to improve mental health.

## Data Availability

The data used and analyzed in this study are available from the corresponding author upon reasonable request.

## Abbreviations

COVID-19: Coronavirus disease 2019
PHQ-9: Patient Health Questionnaire-9
PDI: Peritraumatic Distress Inventory
